# Emotional Intelligence Training Correlates With Medical Students’ Apprehension of AI in Healthcare: A Single-Institution, Observational Study

**DOI:** 10.7759/cureus.94349

**Published:** 2025-10-11

**Authors:** Austin P Runde, Shambhavi Mishra, Marina Feffer, Ramzan Shahid

**Affiliations:** 1 Medicine, Loyola University Chicago Stritch School of Medicine, Maywood, USA; 2 Biostatistics Collaborative Core, Clinical Research Office, Loyola University Chicago Stritch School of Medicine, Maywood, USA; 3 Pediatrics, Loyola University Medical Center, Maywood, USA

**Keywords:** artificial intelligence (ai), doctor-patient relationship, emotional intelligence (ei), healthcare technology, likert-type scale, medical education, survey

## Abstract

Background: The use of artificial intelligence (AI) in healthcare is becoming increasingly widespread. While AI has been touted to improve many aspects of the field, its potential to undermine the doctor-patient relationship has also been recognized. It is not known what future physicians believe regarding the potential effects of AI on physicians' professional relationships. It is also unknown whether formal training in emotional intelligence (EI) and resilience strategies influences medical students' opinions on the use of AI in healthcare.

Objective: To ascertain medical students’ opinion on the potential impact of AI on various EI-related aspects of healthcare, such as its use in guiding doctor-patient interactions, and to determine whether formal training in emotional intelligence and resilience (EIR) is associated with their opinion on these topics.

Methods: Approximately 700 medical students were asked to voluntarily and anonymously complete a 12-item survey. All survey items were required to be answered for students' responses to be accepted and analyzed. Agreement with survey items was measured via a Likert-type scale, with 1 = strongly disagree and 5 = strongly agree. Results were summarized as means and standard deviations, stratified by whether students did (EIR^+^) or did not (EIR^-^) take our institution's EIR elective course. Median responses per survey item were compared across EIR^+/-^ groups via the Mann-Whitney U test (α < 0.05), a non-parametric test comparing two independent samples allowing for unequal group size.

Results: A total of 50 EIR^+^ students (59%) and 35 EIR^-^ students (41%) replied, making the survey response rate approximately 12.14%. Compared to their counterparts, EIR^+^ students indicated statistically significantly greater disagreement with “I believe AI technology will improve the doctor-patient relationship” (3.0 (± 1.2) vs. 3.5 (± 1.0); p = 0.0327). While not statistically significant, EIR^+^ students also reported a similar magnitude (~0.5 points) of greater disagreement with “AI has a place in guiding physicians on how to better interact with their patients and colleagues” (2.9 (± 1.2) vs. 3.4 (± 1.1); p = 0.0701) and “Overall, I think adopting the use of AI in healthcare will be beneficial” (3.6 (± 1.2) vs. 4.0 (± 1.1); p = 0.0521).

Conclusions: The preliminary data from our single-institution, observational study suggest EIR-trained medical students may be more cautious of AI usage in healthcare due to the potential negative impact AI can have on the doctor-patient relationship. However, larger studies using validated surveys are required to confirm this conclusion.

## Introduction

Artificial intelligence (AI) is the ability of a computer system to not only perform tasks that typically require a human operator, but also to learn and make predictions from users’ inputs [1,2]. Modern AI is largely attributed to the 1950s work of Alan Turing and research launched at the Dartmouth Summer Research Project on Artificial Intelligence in 1956 [3-5]. Today, AI has an array of commercial uses, including applications in healthcare, aviation, commerce, education, law enforcement, national security, internet technology, politics, and even space exploration [6]. While some of the large language models (LLMs), such as OpenAI’s ChatGPT and Google’s Gemini, only became freely accessible around 2022-2023, commercial use of AI is far less recent [7,8]. Hospitals have been using AI in the form of clinical decision support (CDS) systems for over 20 years [9]. CDS systems offer treatment recommendations based on real-time clinical metrics, but do not engage with users like LLMs do. Additionally, the National Aeronautics and Space Administration (NASA) has been using AI to plan and troubleshoot missions for decades [10]. While AI implementation has been explored in virtually all fields, the large-scale adoption of AI in healthcare is a particularly intriguing argument with clear pros and cons [11-16].

A fundamental drawback to AI is the absence of human-human interaction and emotional connection. The bond between physicians and patients, the doctor-patient relationship, is inherently therapeutic but requires an empathetic physician to be truly healing. Coined in 1995 by Dr. Daniel Goleman, “emotional intelligence” (EI) broadly refers to a person’s ability to perceive the emotions of themselves and others while expressing/regulating their own [17,18]. In addition to improved interpersonal relationships, EI is positively associated with greater efficiency, reduced burnout, and improved well-being in almost any workplace [19]. In healthcare specifically, EI is a recognized safeguard against depression and is associated with improved interprofessional and provider-patient relationships [18,20-27]. In recognizing the value of EI, several emotional quotient-measuring tools, like the Emotional Quotient Inventory 2.0 (EQ-i 2.0^®^), have even been developed for commercial use [28,29]. While LLMs can, somewhat impressively, emulate the speech pattern and empathy of a human provider, LLMs are not human and thus cannot possess the EI required for true healing [30-40]. EI is an abstract concept unique to humanity. While it is likely that most people, regardless of profession, would agree that EI cannot be fully realized with an AI model, it is not known if or how future physicians believe AI will impact physicians’ own EI and professional relationships, including the doctor-patient relationship.

The objective of our study was two-fold: first, we sought to ascertain medical students’ opinions on the potential impact of AI on various EI-related aspects of healthcare, such as its use in guiding doctor-patient interactions; and second, to determine whether formal training in EI and resilience strategies was associated with students’ opinions on these topics. From a medical education standpoint, understanding medical students' opinions on the use of AI in healthcare is critical because it can inform medical educators of a potentially under-recognized yet increasingly relevant educational need: teaching future doctors how to use AI without compromising their own EI.

We hypothesized that medical students formally trained in emotional intelligence and resilience (EIR) would have a more profound understanding of EI and appreciation for its role in healthcare, and thus be less likely to believe AI will benefit physicians’ EI. To investigate our hypothesis, we created a 12-item, Likert-type survey to ascertain our students' perspectives. Responses were stratified based on whether students had completed our institution's EIR elective course.

This work was presented as a Power Talk at the Future of Pediatric Practice 2025 (Florida Chapter of the American Academy of Pediatrics) in Lake Buena Vista, FL, USA.

## Materials and methods

EIR course logistics

The EIR elective at Loyola University Chicago (LUC) Stritch School of Medicine (SSOM) is offered annually to second-year (M2) students and consists of six, two-hour-long sessions occurring monthly during the academic year. Of the ~175 medical students total in each class, typically 25-35 students enroll in and complete the elective. The course is designed to teach EI as a foundation for developing strong communication and interpersonal skills [41]. Recognizing EI as a key component in establishing rapport and trust with patients, the course also emphasizes its importance in leadership development. In addition to these core competencies, students are educated on applying EI skills to build personal resilience to maintain EI skills and mitigate the risk of burnout. The curriculum includes discussion of specific EIR strategies, with opportunities for students to explore and implement techniques aimed at enhancing their own well-being.

AI-EI survey creation

The authors created a 12-item, Likert-type survey to assess students' opinions on the intersection of various AI- and EI-related topics in healthcare and medical education. Survey items were developed at the discretion of the research team as best representing the concepts of our institution's EIR elective course and its relation to AI. For surveys to be accepted and analyzed, all items were required to be answered to prevent skewing due to missing responses.

Study approval and data collection

The current study (#219038) was reviewed and determined to be exempt by the Institutional Review Board (IRB) of LUC. Allopathic medical students from the LUC SSOM classes of 2025, 2026, 2027, and 2028 (the entire student body) were contacted via email and asked to complete the survey, and the following was explicitly indicated in the consent letter: participation is voluntary, any data collected is for research purposes only and will not affect academic standing, and data collected may be used for publication but will be de-identified in any article or presentation in which it is used.

Statistical analysis

Approximately 700 students were invited. A total of 85 students completed the survey and were included in this analysis. Responses to each survey item were scored on a Likert-type scale indicating level of agreement with each of the 12 statements (1 = strongly disagree, 2 = disagree, 3 = neutral, 4 = agree, and 5 = strongly agree). Results were summarized as means and standard deviations, as well as medians and interquartile ranges, stratified by whether students did (EIR^+^) or did not (EIR^-^ (control)) complete the EIR elective during their second year of medical school. Median responses per survey item are compared across EIR^+/-^ groups via the Mann-Whitney U test, a non-parametric test comparing two independent samples, allowing for unequal group size. Tests are two-sided with a threshold for significance of α < 0.05. Correction for multiple hypothesis testing was not applied due to the limited sample size and subtle expected effects, which could have led to overcorrection and loss of interpretability. All analyses were completed via Stata^®^ statistical software v.18 (StataCorp LLC, College Station, TX) [42].

## Results

Of the ~700 students invited, 85 replied to the survey (response rate ≈ 12.14%) and were included in the statistical analysis (Table 1). Of those who replied, 50 (59%) were EIR^+^ students and 35 (41%) were EIR^-^ students.

**Table 1:** Results of survey and statistical analysis. Means and standard deviations (SD), as well as medians (Mdn) and interquartile ranges (IQR), are displayed for each survey item, stratified by emotional intelligence and resilience (EIR) completion status. Data from the Mann-Whitney U test comparing median scores across EIR^+/-^ groups are listed (*^U ^*= corresponds to Mann-Whitney U test comparing median scores across EIR^+/-^ groups; * = p < 0.05). EI: emotional intelligence.

Survey items	EIR^+ ^(n = 50)		EIR^- ^(n = 35)		p-value*^U^*	z-statistic*^U^*
							Mean ± SD	Mdn (IQR)	Mean ± SD	Mdn (IQR)
							1. I will use AI in my future practice to assist with, or even handle some of, the tasks required of today’s physician, such as responding to MyChart^®^ messages and writing after-visit notes.	3.7 ± 1.2	4 (3-5)	3.8 ± 1.1	4 (3-5)	0.8966	0.13
	2. AI has a place in guiding physicians on how to better interact with their patients and colleagues.	2.9 ± 1.2	3 (2-4)	3.4 ± 1.1			3 (3-4)	0.0701	1.811
3. I would be okay if my own physician had used AI to “coach” them on how to be more empathetic towards you at your appointment.	2.8 ± 1.3	3 (2-4)	3.1 ± 1.3	3 (2-4)	0.2566	1.135
4. I support physicians using AI to advise them on how to deliver bad news and broach sensitive topics.	3.1 ± 1.2	3 (2-4)	3.3 ± 1.3	3 (2-4)	0.5355	0.62
5. There is utility for AI in helping physicians identify and analyze their emotions to better process difficult situations. For example, providing physicians with a dedicated interface to “converse” with following the death of a patient.	3.0 ± 1.2	3 (2-4)	3.0 ± 1.3	3 (2-4)	0.7556	-0.311
6. I believe AI technology will improve the doctor-patient relationship.	3.0 ± 1.2	3 (2-4)	3.5 ± 1.0	3 (3-4)	0.0327*	2.136
7. Overall, I think adopting the use of AI in healthcare will be beneficial.	3.6 ± 1.2	4 (3-4)	4.0 ± 1.1	4 (4-5)	0.0521	1.942
8. I believe EI skills such as empathy, communication, and listening are critical for physicians, especially as AI technology becomes commonplace.	4.7 ± 0.7	5 (5-5)	4.8 ± 0.6	5 (5-5)	0.7329	0.341
9. It is essential to teach innately human skills (e.g., EI skills) to medical students as more AI technology is used.	4.7 ± 0.8	5 (5-5)	4.7 ± 0.6	5 (5-5)	0.6714	0.424
10. I believe AI technology will augment the empathetic care that doctors provide by allowing more time with their patients.	3.5 ± 1.2	3.5 (3-5)	3.7 ± 1.3	4 (3-5)	0.4344	0.782
11. I am concerned that the use of AI technology will cause attrition of critical human skills that physicians need.	3.6 ± 1.2	4 (3-5)	3.4 ± 1.2	4 (2-4)	0.3636	-0.908
12. I believe AI technology will hurt the doctor-patient relationship.	2.9 ± 1.1	3 (2-4)	2.6 ± 1.1	2 (2-3)	0.1713	-1.368

EIR^+^ students reported statistically significantly greater disagreement with survey item 6, “I believe AI technology will improve the doctor-patient relationship,” than students in the EIR^-^ group (p = 0.0327).

While not statistically significant, EIR^+^ students reported a similar magnitude of greater disagreement versus EIR^-^ counterparts with items 2 and 7, “AI has a place in guiding physicians on how to better interact with their patients and colleagues” and “Overall, I think adopting the use of AI in healthcare will be beneficial,” respectively. There was a difference of nearly 0.5 points between groups for both items (∆0.5 and ∆0.4, respectively). For item 2, EIR^+^ students replied with a mean of 2.9 (± 1.2) and EIR^-^ counterparts replied with a mean of 3.4 (± 1.1; p = 0.0701). For item 7, EIR^+^ students replied with a mean of 3.6 (± 1.2) and EIR^-^ counterparts replied with a mean of 4.0 (± 1.1; p = 0.0521).

Average responses between EIR^+^ and EIR^-^ students did not display significant/trends toward differences for survey items 1, 3-5, and 8-12.

## Discussion

Study commentary

The preliminary data from this single-institution, observational study suggest medical students trained in EIR are less likely to believe AI will improve the doctor-patient relationship compared to untrained counterparts. Our impression is that these data may reflect the enhanced understanding of EI acquired by elective participants; students who completed the EIR elective might consequently be more apprehensive of AI’s potential impact on the doctor-patient relationship. However, this conclusion requires confirmation with larger studies using validated surveys. And while EIR^+^ students indicated they are less likely to believe AI will improve the doctor-patient relationship, this cannot be interpreted as they believe AI will harm the doctor-patient relationship; the two statements are not interchangeable, hence why both items were utilized in our survey.

As previously mentioned, the argument for adopting AI in healthcare has clear pros and cons [43].

Firstly, AI has been used by many health systems for a substantial period of time, thus making future implementation feasible.

Secondly, patient outcomes are typically the direct result of a human provider’s decision(s). As it is well known how burnout, exhaustion, stress, emotion, and decision fatigue can all contribute to medical mistakes, using AI to affirm a provider’s decision could be a welcome development for health systems, providers, and patients alike from legal, financial, and ethical perspectives. For example, knowing AI agreed with a provider amid a bad outcome may mitigate guilt (legal and moral) borne by the healthcare team; though, in turn, AI could very well implicate the team should it disagree.

Thirdly, AI could be used to identify subtle patterns that even the most adept clinicians and researchers would otherwise miss, such as outcome risk factors and public health trends [44-46]. Following the COVID-19 pandemic, there has been significant interest from global agencies in using AI to predict future pandemics [47,48].

And fourthly, owing to its ability to transcribe and proofread, AI has been argued to help reduce provider burnout due to time spent on electronic medical records [49,50]. However, advocating for AI in healthcare is fraught with a significant concern inherent to the use of this software. While AI has been touted as a low-risk yet highly effective solution to many of healthcare’s most significant problems, the potential of AI to undermine one of the most revered, fundamental aspects of medicine, the doctor-patient relationship, has also been recognized [37,40,51-58]. It remains a question whether the pros of AI adoption outweigh the possible detriment AI could have on the doctor-patient relationship, or whether AI can be safely integrated without overshadowing the doctor-patient relationship altogether.

Study limitations

Though preliminary/exploratory, single-institution, and observational in nature, we are excited to present novel data characterizing both medical students’ perception of the potential impact of AI on physicians’ EI and the effect EIR training may have on their perception. However, our study has several limitations.

Firstly, we acknowledge the small sample size of our response cohort as well as the imbalance within it. We received 85 responses, only 41% of which were control group (EIR^-^) students. Small sample size inherently reduces the generalizability and increases the variability of results, which we acknowledge as a limitation of our study. While the Mann-Whitney U test does not require equal group sizes, it is prudent to recognize that any discrepancy between the number of control and experimental group responses is inherently a source of bias, even if mitigated by the statistical test employed.

Secondly, we recognize the selection biases that could be present in our study. As with any survey-type study, there is an inherent selection bias regarding those who respond to the survey; students who respond may have stronger feelings regarding the research topic and thus may be more motivated to reply. Similarly, as seats in our EIR elective are secured on a first-come/first-serve basis, each cohort is self-selected. Thus, EIR participants may have a stronger interest in EI and be more adamant in and likely to express their views compared to those who did not take the course.

Thirdly, we acknowledge that the authors of this study are part of the EIR course leadership. While none of the authors responded to the survey nor intentionally attempted to influence students' AI/EI opinions in any capacity, we would be remiss if we failed to acknowledge this relationship as a potential source of bias. While the authors gave their best efforts to ensure their opinions were not disclosed in or outside the EIR course, we must, in good faith, acknowledge the reality of unconscious influence and how this could unintentionally impact course instruction, framing of survey questions, and survey results.

And fourthly, we acknowledge our survey is not a validated screening questionnaire. This survey was created by the authors using their best judgment to assess students’ perceptions in a balanced, non-leading manner. We appreciate the responsibility of survey authors and interviewers to frame questions in a manner that best ensures objectivity and reduces bias [59]. We would create a validated questionnaire for future surveys on the relationship between AI and EI, and methods to create validated questionnaires for other AI-related topics have been described [60-64].

Current literature

To our knowledge, only one other study has directly surveyed students regarding the intersection between AI and EI. The results of our study contrast with those from Ghotbi's study of students (n = 235) at an international university in Japan [65]. Students were asked to write an essay detailing their opinion on “emotional AI,” which is the use of AI to help people better understand and regulate their emotions. Qualitative analysis of the essays indicated that most students (137, 58%) had a positive view of emotional AI. Supporting reasons cited included "improved mental health triaging," "increased employee/customer satisfaction," and "enhanced understanding of human emotions." Interestingly, “inability to compensate for human interaction” and “lack of affection and morality” were listed as reasons why students disfavored emotional AI, themes that echo our concern regarding the use of AI in healthcare.

While not addressing the relationship between AI and EI directly, several other surveys and interviews have been conducted to ascertain students’ and educators’ opinions on AI. Results were balanced in a study of undergraduate students and professors at Wenzhou University in Wenzhou, Zhejiang, China [66]. Respondents expressed both support and concern for the use of AI in education. Interestingly, students and teachers suggested AI could both aid and impede the different emotional components of learning. While respondents indicated the “always-available” nature of AI reduced anxiety related to learning difficulties and improved student support, they were simultaneously concerned about reliance on AI and how this could detract from valuable interpersonal interactions. Other recent surveys, including students and musicians, have also revealed overall positive views on AI globally [67-70].

Future directions

In terms of future research, we would like to further ascertain our students’ specific reasons for supporting or opposing the adoption of AI in healthcare. We would also like to greatly increase our sample size by surveying medical students at other institutions. And while EI training benefits all physicians, it is likely to be particularly beneficial in specialties where a large fraction of patients may be unable to clearly articulate concerns, such as in pediatrics and psychiatry [71]. As such, we would like to see if physicians’ specialty is associated with their opinion on AI usage in healthcare.

## Conclusions

Our findings suggest EIR training is associated with greater apprehension toward the use of AI in healthcare and its ability to improve the doctor-patient relationship. However, larger studies with validated surveys are required to confirm this finding. Regardless, we believe the best way to protect the doctor-patient relationship is not to bar AI from healthcare, but rather to thoroughly educate healthcare providers and patients alike on its benefits and pitfalls. Like a CT scan or set of labs, AI is merely a tool that should be used to complement a physician’s assessment of the entire clinical picture.
